# Epigenetic and Post‐Transcriptional Regulation of Autophagy in Inflammatory Bowel Disease

**DOI:** 10.1155/grp/7973438

**Published:** 2026-11-08

**Authors:** Yujun Liu, Wenliang Jiang, Jiaxin Zhou, Feng Xun, Haifeng Li, Yujiang Li, Jie Zhang, Yun Qian, Honggang Wang

**Affiliations:** ^1^ Department of General Surgery The Affiliated Taizhou People′s Hospital of Nanjing Medical University Taizhou School of Clinical Medicine Nanjing Medical University Taizhou Jiangsu China njmu.edu.cn; ^2^ Department of the Second Clinical Medical School Nanjing Medical University Nanjing Jiangsu China njmu.edu.cn; ^3^ Department of General Surgery Postgraduate Training Base of Dalian Medical University (Taizhou People′s Hospital) Taizhou Jiangsu China; ^4^ Department of Gastroenterology The Affiliated Taizhou People′s Hospital of Nanjing Medical University Taizhou School of Clinical Medicine Nanjing Medical University Taizhou Jiangsu China njmu.edu.cn

**Keywords:** autophagy-related genes, chromatin remodelling, DNA methylation, epigenetic regulation, histone modifications, inflammatory bowel disease, noncoding RNAs, post-transcriptional regulation

## Abstract

Inflammatory bowel disease (IBD), principally comprising Crohn’s disease (CD) and ulcerative colitis (UC), encompasses chronic immune‐mediated inflammatory disorders of the gastrointestinal tract. Genome‐wide association studies have identified hundreds of susceptibility loci associated with IBD. Recent studies have highlighted the critical importance of autophagy in maintaining intestinal epithelial homeostasis, regulating host–microbiota interactions, coordinating immune responses and restricting microbial pathogens. Furthermore, emerging evidence indicates that classical epigenetic mechanisms and post‐transcriptional regulators may influence autophagy‐related pathways in IBD and thereby shape intestinal inflammatory responses. This review critically evaluates current evidence linking epigenetic and post‐transcriptional regulation to autophagy in IBD, distinguishes established mechanisms from indirect associations and discusses their mechanistic and translational implications.

## 1. Introduction

Inflammatory bowel disease (IBD) comprises chronic immune‐mediated inflammatory disorders of the gastrointestinal tract, principally Crohn’s disease (CD) and ulcerative colitis (UC). Clinical manifestations include abdominal pain, diarrhoea, urgency and other symptoms that vary with disease location and activity [1]. IBD often develops in adolescence or early adulthood and can substantially impair quality of life [2]. Its pathogenesis reflects interactions among genetic susceptibility, mucosal immune dysregulation, epithelial barrier dysfunction, the gut microbiota and environmental and metabolic factors [3].

Autophagy is a lysosome‐dependent degradative and recycling pathway that supports cellular quality control, metabolic adaptation, stress responses and immune regulation [4, 5]. Macroautophagy, microautophagy and chaperone‐mediated autophagy are the three principal forms [4]; this review focuses mainly on macroautophagy and related LC3‐lipidation processes relevant to intestinal biology. In the intestine, autophagy contributes to epithelial homeostasis, antimicrobial peptide secretion, microbial handling and the regulation of innate and adaptive immunity [6–8]. Its dysregulation can therefore promote persistent mucosal inflammation through impaired barrier and host–defence functions [7, 8].

Classical epigenetic regulation refers here to chromatin‐based control of gene expression mediated by DNA methylation, covalent histone modifications and ATP‐dependent chromatin remodelling without alteration of the DNA sequence. By contrast, most miRNAs, lncRNAs, circRNAs and tRNA‐derived small RNAs discussed in this review act mainly through post‐transcriptional mechanisms, including mRNA repression, altered transcript stability, competing endogenous RNA (ceRNA) networks, miRNA processing and RNA‐protein interactions. Accumulating evidence suggests that both classical epigenetic mechanisms and post‐transcriptional regulators can influence autophagy‐related pathways relevant to IBD, although the strength of evidence varies substantially among regulatory mechanisms. Defining these relationships may improve mechanistic understanding of IBD and identify candidate biomarkers or therapeutic pathways requiring further validation.

Previous reviews have provided important summaries of autophagy dysfunction in IBD, epigenetic alterations associated with intestinal inflammation or selected noncoding‐RNA mechanisms [6–10]. However, these areas have often been discussed separately, and the evidence linking specific classical epigenetic or post‐transcriptional regulators to autophagy‐related genes (ATGs) and pathways in IBD remains dispersed across genetic, molecular, cellular and disease‐focused studies. In particular, the extent to which these regulatory relationships have been validated in human IBD, experimental colitis or disease‐relevant cellular models is not always clear.

In this review, we therefore focus on the intersection between epigenetic or post‐transcriptional regulation and autophagy in IBD. We organise current evidence according to major regulatory mechanisms, including DNA methylation, histone modification, chromatin remodelling and noncoding RNAs, and examine how they may influence IBD‐related autophagy genes and pathways. Particular attention is given to the source and strength of the available evidence, distinguishing findings from human IBD studies, experimental colitis models, disease‐relevant cellular systems and mechanisms inferred from other disease contexts. Through this framework, we aim to clarify which regulator‐autophagy interactions are supported in IBD and which remain potential mechanisms requiring further validation, while also considering their implications for biomarker development and therapeutic intervention.

## 2. Literature Search and Selection

This review was based on a structured PubMed literature search conducted during manuscript preparation and updated during revision, with the final targeted update completed on 24 September 2026. Relevant studies were identified through searches of PubMed, supplemented by screening the reference lists of relevant original studies and review articles. The main search terms included combinations of “inflammatory bowel disease”, “Crohn′s disease”, “ulcerative colitis”, “autophagy”, “autophagy‐related genes”, “ATG16L1”, “IRGM”, “NOD2”, “DNA methylation”, “histone modification”, “chromatin remodelling”, “microRNA”, “lncRNA”, “circRNA”, “tRNA‐derived small RNA” and “post‐transcriptional regulation”. The final targeted translational update additionally combined IBD‐related and epigenetic/RNA‐related terms with “biomarker”, “treatment response”, “therapy”, “RNA‐based therapy” and “delivery”; autophagy‐related terms were added where appropriate to identify mechanism‐linked translational studies. The search strategy was designed to support a structured narrative synthesis rather than an exhaustive systematic review.

No lower publication‐date limit was imposed because several earlier studies provide important genetic and mechanistic background for IBD‐associated autophagy pathways. More recent studies were prioritised when evaluating current mechanistic and translational evidence, while earlier landmark studies were retained where necessary.

For the mechanistic synthesis, studies were included when they examined a classical epigenetic mechanism or a noncoding‐RNA‐mediated post‐transcriptional mechanism in relation to autophagy in human IBD tissues, patient cohorts, clinical samples, experimental colitis models, intestinal epithelial cells, immune‐cell systems, organoids or other disease‐relevant models. When direct IBD evidence was unavailable, studies from cancer, neurodegenerative disease or general cell‐biological contexts were considered only when they provided essential evidence for a specific regulator‐autophagy relationship and were explicitly interpreted as indirect evidence. For the translational sections, clinically relevant studies of epigenetic or RNA‐based biomarkers, therapeutic modulation, treatment‐response prediction or delivery strategies in IBD were also considered even when autophagy dependence had not been demonstrated; such studies were explicitly presented as broader translational evidence rather than as evidence for an established autophagy‐dependent mechanism. Studies were excluded when they lacked sufficient relevance to the mechanistic or translational scope of the review, duplicated a more complete report of the same study, lacked sufficient methodological or mechanistic information for interpretation or consisted solely of editorials or commentaries. Purely computational predictions were not treated as established mechanistic evidence unless explicitly retained as hypothesis‐generating findings. Conference abstracts were screened when necessary to assess the currency of ongoing therapeutic development but were not used as definitive mechanistic or efficacy evidence.

Screening proceeded in two stages: titles and abstracts were first assessed for relevance, and potentially relevant full texts were then evaluated for disease context, model type, target validation, autophagy assessment where applicable, rescue or intervention evidence and IBD relevance. For the mechanistic synthesis, evidence was described as direct IBD evidence, disease‐relevant experimental evidence or indirect non‐IBD mechanistic evidence; purely computational or predicted networks retained for context were labelled hypothesis‐generating evidence. These labels describe evidentiary context and directness of disease relevance rather than formal study‐quality grades. Because the aim was a structured narrative synthesis rather than a systematic review, formal systematic‐review procedures such as a PRISMA flow diagram, risk‐of‐bias assessment and quantitative evidence grading were not applied.

## 3. Autophagy and IBD

Autophagy is dynamically regulated by nutrient and energy availability, endoplasmic reticulum stress, oxidative stress, hypoxia and microbial exposure [4, 11]. Autophagy has been shown to remove damaged organelles, protein aggregates, invading microorganisms and other cytoplasmic cargo to prevent cellular stress and inflammation [4, 5]. Autophagy also regulates innate immune signalling and inflammatory responses, which is crucial for maintaining intestinal immunity. During infection, autophagy contributes to microbial restriction while modulating inflammatory signalling [5, 12]. Moreover, autophagy influences the expression and release of inflammatory mediators, for instance, by mitigating the inflammatory response through the downregulation of proinflammatory mediators such as interleukin‐1*β* (IL‐1*β*) and tumour necrosis factor‐*α* (TNF‐*α*) [5, 12]. Autophagy can also restrain inflammasome activation and epithelial pyroptosis, although the direction and pathological consequence of autophagy are context dependent [4, 5, 12]. Specific noncoding RNA–autophagy mechanisms are discussed in Section 4.4. Genetic studies first highlighted a direct connection between autophagy‐related pathways and IBD susceptibility. Variants in ATGs contribute to genetic susceptibility to CD [13, 14].

### 3.1. ATGs and IBD

In IBD, autophagy‐related pathways influence antimicrobial defence, epithelial homeostasis and innate and adaptive immune responses. ATGs participate in antimicrobial selective autophagy and other autophagy‐related host–defence processes and the regulation of microtubule‐associated protein 1 light chain 3 (LC3)‐associated phagocytosis [4, 15]. During autophagosome formation, cytosolic LC3‐I is conjugated to phosphatidylethanolamine to generate membrane‐associated LC3‐II. After autophagy activation, LC3 lipidation facilitates autophagic membrane expansion and cargo sequestration and also participates in noncanonical processes such as LC3‐associated phagocytosis [4, 15]. Several susceptibility genes implicated in IBD converge on pathways involved in innate immunity and autophagy‐mediated host defence. Genome‐wide association and related genetic studies have identified ATG16L1 and IRGM as susceptibility genes associated with IBD. In addition, polymorphisms in NOD2 have been shown to increase susceptibility, particularly to CD [13, 14, 16].

ATG16L1 is a core component of the autophagy machinery and forms a complex with the ATG12–ATG5 conjugate. The ATG12–ATG5–ATG16L1 complex functions as an E3‐like factor that facilitates LC3 lipidation at autophagic membranes, a key step in autophagosome formation. Through this function, ATG16L1 contributes not only to canonical autophagy but also to autophagy‐related host–defence processes that are particularly relevant to intestinal homeostasis [17].

Genetic studies have identified ATG16L1 as an important susceptibility locus for CD. Among the reported variants, rs2241880 is one of the most extensively studied and results in a threonine‐to‐alanine substitution at amino acid 300 of ATG16L1, commonly referred to as the T300A variant. Population studies have consistently linked rs2241880/T300A to CD susceptibility, although the magnitude and direction of the association vary among ethnic groups and clinical phenotypes [13, 18].

Functionally, T300A can alter ATG16L1‐dependent autophagy‐related responses, including microbial handling, inflammatory signalling and LC3‐associated phagocytosis. Importantly, the consequences of this variant should not be regarded as uniformly detrimental. Recent studies indicate that its functional effects may depend on allele dosage and biological context. Heterozygous and homozygous carriers can display different inflammatory and host–defence phenotypes, suggesting that T300A modifies rather than simply abolishes ATG16L1 function [19].

These findings place ATG16L1 at the interface between genetic susceptibility, autophagy‐related host defence and intestinal inflammation. However, rs2241880/T300A represents a functional risk variant rather than a complete loss of ATG16L1 activity. Therefore, statements implying that the variant invariably prevents autophagosome formation or produces a uniform inflammatory phenotype should be avoided. The contribution of ATG16L1 variation to CD is more appropriately understood as context dependent and shaped by genetic background, cell type, microbial exposure and inflammatory state [19].

Human IRGM is closely associated with autophagy and has been implicated in the control of pathogen‐directed responses and excessive inflammatory signalling. Jena et al. showed that human IRGM suppresses cyclic GMP–AMP synthase (cGAS)‐stimulator of interferon genes (STING) and retinoic acid‐inducible gene I (RIG‐I)‐mitochondrial antiviral signalling protein (MAVS) pathways, thereby limiting excessive interferon signalling [20].

Human IRGM also regulates inflammasome activation. Mehto et al. demonstrated that human IRGM restricts NLRP3 inflammasome activation by interfering with inflammasome assembly and promoting SQSTM1/p62‐dependent selective autophagy [21, 22]. These findings indicate that human IRGM can couple selective autophagic removal of inflammasome components with the control of excessive inflammatory signalling.

Murine Irgm proteins also participate in host responses to bacterial stimuli, but the available murine evidence should be interpreted separately from the human IRGM pathway. Finethy et al. showed that the loss of murine Irgm2 enhanced caspase‐11‐dependent inflammatory responses following exposure to lipopolysaccharide, bacterial outer‐membrane vesicles or Gram‐negative bacteria [23]. Taylor et al. demonstrated that murine Irgm1 deficiency impaired myeloid‐cell function in the colonic lamina propria and increased susceptibility to *Citrobacter rodentium* infection [24]. Together, these studies support a role for murine Irgm1 in intestinal myeloid host defence and for murine Irgm2 in restraining caspase‐11‐dependent inflammatory responses. Because primate and murine IRG repertoires have undergone lineage‐specific evolution, these murine findings should not be interpreted as demonstrating one‐to‐one orthology or direct functional equivalence with human IRGM.

Microbial factors can also suppress host autophagy independently of genetic variation in human IRGM. A well‐characterised example is the regulation of intestinal epithelial autophagy by CD‐associated adherent‐invasive *Escherichia coli* (AIEC) through host microRNAs (miRNAs) [25, 26]. Because this mechanism represents post‐transcriptional regulation of host autophagy rather than an IRGM genetic mechanism, it is discussed separately in the noncoding RNA section below.

Nucleotide‐binding oligomerisation domain‐containing protein 2 (NOD2) is a cytosolic pattern‐recognition receptor that senses bacterial muramyl dipeptide and links microbial recognition to inflammatory signalling and antibacterial autophagy [27]. Upon muramyl‐dipeptide recognition, NOD2 activates downstream receptor‐interacting serine/threonine‐protein kinase 2 (RIPK2), NF‐*κ*B and mitogen‐activated protein kinase (MAPK) signalling and can recruit ATG16L1 to bacterial entry sites to promote antibacterial autophagy [27, 28]. NOD2 dysfunction can impair antimicrobial responses in intestinal epithelial and Paneth‐cell compartments and compromise microbial handling [7, 27, 29]. Among the genetic variants associated with IBD, NOD2 variants are among the strongest common genetic risk factors for ileal CD, although the magnitude of risk depends on the specific variant and allele dosage [16]. The autophagy‐related roles of ATG16L1, human IRGM and NOD2, together with the species‐specific host–defence functions of murine Irgm proteins, are summarised in Figure 1.

**Figure 1:**
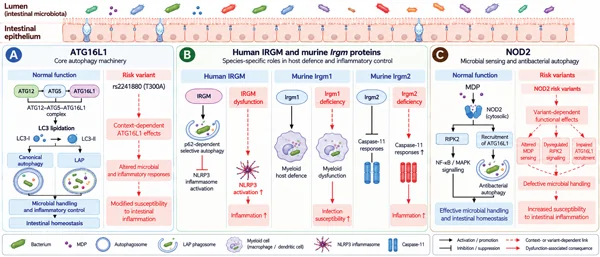
Simplified overview of autophagy‐related IBD susceptibility pathways. (A) ATG16L1 functions with the ATG12‐ATG5 conjugate to support LC3 lipidation, canonical autophagy, LC3‐associated phagocytosis (LAP) and microbial handling. The rs2241880/T300A risk variant is shown as exerting context‐dependent effects on ATG16L1‐dependent responses rather than causing uniform loss of function. (B) Species‐specific functions of human IRGM and murine Irgm proteins are presented separately. Human IRGM promotes SQSTM1/p62‐dependent selective autophagic control of NLRP3 inflammasome activation; murine Irgm1 is linked primarily to intestinal myeloid host defence, whereas murine Irgm2 restrains caspase‐11‐dependent inflammatory responses. Murine Irgm1 and Irgm2 are not depicted as one‐to‐one orthologues or direct functional equivalents of human IRGM. (C) Bacterial muramyl dipeptide (MDP) activates NOD2, leading to RIPK2‐NF‐*κ*B/MAPK signalling and ATG16L1 recruitment to bacterial entry sites, thereby supporting antibacterial autophagy and microbial handling. NOD2 risk variants are shown as producing variant‐dependent effects on MDP sensing, RIPK2 signalling, and ATG16L1 recruitment that may converge on defective microbial handling and increased susceptibility to intestinal inflammation. Solid black arrows indicate activation or promotion; blunt‐ended black lines indicate inhibition or suppression; solid red arrows indicate dysfunction‐associated consequences; dashed red arrows indicate context‐ or variant‐dependent links. The pathways summarised in the figure derive from different epithelial and immune‐cell populations and experimental settings and should not be interpreted as occurring simultaneously within a single cell type. Abbreviations: caspase‐11, caspase 11; LAP, LC3‐associated phagocytosis; LC3, microtubule‐associated protein 1 light chain 3; MAPK, mitogen‐activated protein kinase; MDP, muramyl dipeptide; NF‐*κ*B, nuclear factor‐*κ*B; NOD2, nucleotide‐binding oligomerisation domain‐containing protein 2; NLRP3, NLR family pyrin domain containing 3; RIPK2, receptor‐interacting serine/threonine‐protein kinase 2; SQSTM1/p62, sequestosome 1/p62.

### 3.2. The Role of Autophagy in Regulating Inflammatory Responses in IBD

Autophagy influences intestinal inflammation through effects on immune‐cell activation, antigen processing, cellular metabolism, cell survival and apoptotic‐cell clearance [5, 7, 30]. Autophagy can modulate antigen processing and presentation in antigen‐presenting cells, although the direction of this effect depends on the autophagy pathway, antigen source and cell type [31]. Autophagy also influences the metabolic fitness and functional state of intestinal immune cells [5]. Dysregulated CD4+ T‐cell responses contribute to chronic intestinal inflammation [3]. Autophagy intersects with apoptosis and apoptotic‐cell clearance pathways and can thereby influence the persistence or resolution of intestinal inflammation [32]. In contrast, dysregulation of the autophagy pathway may contribute to aberrant immune‐cell activation and sustained inflammatory responses, thereby contributing to persistent mucosal inflammation in IBD [5, 7].

The gut microbiota interacts bidirectionally with epithelial and immune‐cell autophagy and thereby influences mucosal immune homeostasis [6, 33]. Autophagy can alter epithelial barrier function, microbial clearance and the intestinal environment, thereby indirectly shaping microbial composition and metabolic output [6, 7, 33]. Specifically, autophagy can regulate epithelial barrier integrity and bacterial handling, thereby modifying the ecological conditions experienced by the gut microbiota [6, 7, 33]. In addition, autophagy regulates the ability of IECs to recognize and remove bacteria, thereby affecting the composition of the gut microbiota. Conversely, the gut microbiota affects the regulation of autophagy through the generation of metabolites and interactions with the intestinal immune system [6, 34]. Microbial metabolites, including short‐chain fatty acids, can modulate epithelial and immune‐cell autophagy, although these effects are metabolite‐ and cell‐type‐dependent [34]. Conversely, pathogenic bacteria can manipulate host autophagy to enhance intracellular persistence or alter inflammatory signalling [6, 7, 33]. Autophagy therefore contributes to the reciprocal relationship between epithelial cells and the commensal microbiota and is particularly important for specialised epithelial lineages such as Paneth and goblet cells [29, 33, 35].

Dysregulated innate and adaptive immune responses within the intestinal mucosa are central to the onset and progression of IBD. Paneth cells, which reside in the crypts of the small intestine, are important components of innate mucosal defence. They secrete antimicrobial molecules, including lysozyme, *α*‐defensins and phospholipase A2, and participate in cytokine‐mediated inflammatory networks that influence intestinal homeostasis and inflammation [29]. Goblet cells are distributed throughout both the small and large intestines and contribute to mucosal barrier integrity through mucin secretion. Impaired mucin secretion or disruption of the mucus barrier can increase bacterial contact with epithelial cells, thereby promoting mucosal inflammation and contributing to IBD pathogenesis [35].

## 4. Epigenetic and Post‐Transcriptional Regulation of Autophagy in IBD

Classical epigenetic regulation provides one interface between environmental exposure, inflammatory signalling and persistent changes in cellular function in IBD through DNA methylation, histone modification and chromatin remodelling. In parallel, noncoding RNAs can regulate gene expression post‐transcriptionally through effects on mRNA stability or translation, miRNA availability and RNA–protein interactions. These two regulatory layers can converge on autophagy, epithelial stress responses, microbial handling and immune activation [9, 10]. This convergence is particularly relevant to IBD, in which impaired autophagy may disturb epithelial integrity and antimicrobial defence while altering inflammatory responses in both epithelial and immune cells [7, 9, 10].

However, these relationships have not been established to the same extent across different regulatory classes. For some pathways, classical epigenetic or post‐transcriptional regulation has been directly connected to autophagy and intestinal inflammation in disease‐relevant models. In other cases, IBD‐associated regulatory changes and autophagy‐regulatory mechanisms have been identified separately and have not yet been linked experimentally. The following sections therefore focus on how strongly each proposed regulator‐autophagy relationship can currently be connected to IBD, rather than treating all reported associations as equivalent.

### 4.1. DNA Methylation: From Disease‐Associated Epigenetic Changes to Autophagy

DNA methylation is one of the most stable forms of epigenetic regulation and can produce sustained changes in gene transcription through the activity of DNA methyltransferases, including DNMT1, DNMT3A and DNMT3B [36]. In IBD, disease‐associated alterations in DNA methylation have been identified in both intestinal tissues and peripheral blood samples. Differential promoter methylation has been reported in colonic biopsies from patients with UC, while paediatric and adult IBD cohorts have shown reproducible changes in the circulating methylome, including hypomethylation of RPS6KA2 [37–39]. These findings indicate that altered DNA methylation is a reproducible component of the IBD epigenetic landscape rather than a phenomenon restricted to a single tissue or cohort.

The functional consequences of these methylation changes are likely to be heterogeneous. DNA methylation can influence immune‐cell differentiation and inflammatory transcriptional programs, including macrophage and T‐cell responses [40], indicating that part of its contribution to IBD may occur independently of autophagy. At the same time, experimental studies outside IBD have demonstrated that aberrant DNA methylation can alter the expression of ATGs and modify the expression or activity of ATG5, LC3 and other components of the autophagy machinery [41, 42]. However, these mechanisms have not yet been directly validated in IBD. These observations raise the possibility that persistent methylation changes in intestinal epithelial or immune cells could also modify autophagic capacity during chronic intestinal inflammation.

The key unresolved question is therefore not whether DNA methylation is altered in IBD or whether it can regulate autophagy, since both are supported independently. Rather, it is whether disease‐associated methylation changes in IBD directly reprogram autophagy in specific intestinal cell populations. This distinction is important because a stable methylation change affecting an ATG in intestinal epithelial cells could have very different consequences from the same epigenetic alteration in macrophages or lymphocytes. Future studies integrating cell‐type‐resolved methylation profiles with functional measurements of autophagy will therefore be required to determine whether DNA methylation contributes to persistent defects in epithelial stress tolerance, microbial clearance or inflammatory control in CD and UC.

At present, the DNA methylation–autophagy connection in IBD should be regarded as biologically plausible but mechanistically immature. Human studies provide strong evidence for disease‐associated methylation changes, whereas the direct autophagy‐regulatory component still relies substantially on mechanistic observations from other experimental systems. This makes DNA methylation an important area for targeted validation rather than an established autophagy‐regulatory mechanism in IBD [37–42].

### 4.2. Histone Modifications: A Potential Mechanistic Bridge Between Chromatin State and Autophagy

Histone post‐translational modifications influence chromatin accessibility and transcription and may thereby coordinate inflammatory and autophagy‐related programs [10]. Among the histone‐modifying enzymes implicated in intestinal inflammation, SET domain‐containing protein 2 (SETD2) provides one of the clearest examples of a potential connection between chromatin regulation and autophagy.

SETD2 is the principal methyltransferase responsible for H3K36 trimethylation and participates in transcriptional regulation, RNA processing, genome stability and immune‐cell function [43]. In experimental colitis, SETD2 has been shown to modulate oxidative stress and intestinal inflammatory responses, supporting a role for SETD2‐dependent chromatin regulation in intestinal homeostasis [44]. These observations identify SETD2 as a regulator of oxidative‐stress responses and epithelial adaptation, processes that may intersect with autophagy.

A direct autophagy‐regulatory function of SETD2 has been demonstrated in another disease context. In clear cell renal cell carcinoma, SETD2 deficiency was associated with altered ATG12 regulation and impaired autophagy [45]. When considered together with the colitis findings, these observations suggest that autophagy may represent a previously underexplored mechanism linking SETD2‐dependent chromatin regulation to intestinal stress responses. Importantly, however, the SETD2–ATG12–autophagy axis itself has not yet been directly demonstrated in IBD‐relevant intestinal epithelial or immune‐cell models. Thus, the current evidence identifies a plausible mechanistic bridge rather than a completed disease pathway.

Other histone‐modifying enzymes also support the broader concept that chromatin state can directly influence the autophagic machinery. Sirtuin 6 (SIRT6), for example, has been reported to promote autophagy through direct interaction with Unc‐51‐like autophagy activating kinase 1 (ULK1) and competitive binding to PUMA in non‐IBD experimental systems [46]. These findings reinforce the principle that histone‐modifying enzymes can affect autophagy at the level of pathway regulation, although their contribution to intestinal inflammation remains less well defined than their general autophagic functions.

Compared with DNA methylation, the histone modification field therefore provides a somewhat more specific mechanistic candidate in SETD2, because its intestinal inflammatory function and its autophagy‐regulatory function have both been experimentally demonstrated, albeit in different systems [44, 45]. A key unresolved question is whether these functions converge in intestinal epithelial cells, macrophages, T cells or other disease‐relevant populations. Establishing such convergence would move SETD2 from an emerging candidate to a disease‐specific epigenetic regulator of autophagy in IBD.

### 4.3. Chromatin Remodelling: From BRG1‐Dependent Autophagy to Emerging Candidate Pathways

Chromatin remodelling can alter the accessibility of regulatory DNA regions and thereby reshape transcriptional programs involved in cellular stress responses, inflammation and autophagy [47, 48]. Among the epigenetic mechanisms considered here, chromatin remodelling currently provides one of the more direct examples of how an epigenetic regulator can influence autophagy and intestinal homeostasis.

Brahma‐related gene 1 (BRG1; SMARCA4), an ATPase subunit of the SWI/SNF chromatin‐remodelling complex, represents one of the strongest disease‐relevant examples. In intestinal epithelial cells, BRG1 has been shown to regulate the transcription of several ATGs, including ATG16L1, AMBRA1, ATG7 and WIPI2 [49]. Loss of BRG1 impairs autophagic activity, promotes excessive accumulation of reactive oxygen species, disrupts epithelial homeostasis and aggravates colonic inflammation [49]. This pathway therefore links chromatin accessibility directly to ATG expression and further connects altered autophagy with epithelial stress and intestinal inflammation.

The importance of this finding extends beyond BRG1 itself. It suggests that the disruption of chromatin accessibility can impair autophagy not only through changes in a single autophagy gene but through coordinated transcriptional effects on multiple components of the autophagic machinery. In intestinal epithelial cells, where continuous microbial exposure and metabolic stress require efficient turnover and stress adaptation, such coordinated regulation may be particularly important for maintaining mucosal homeostasis [7, 33, 49].

Other chromatin remodellers have also been linked to autophagy, although their relevance to IBD is less established. CHD6 has been implicated in the coordinated regulation of autophagy and DNA‐damage responses in non‐IBD cellular systems [50], while SQSTM1/p62 has been connected to autophagy‐dependent genome‐maintenance programmes in cancer‐related contexts [51]. These observations suggest that chromatin remodelling and autophagy may intersect through multiple stress‐response pathways. At present, however, these mechanisms are best viewed as candidate extensions of the better‐supported BRG1 paradigm rather than established components of IBD pathogenesis.

The contrast between BRG1 and these candidate regulators illustrates the uneven maturity of the current evidence. BRG1 provides a relatively complete disease‐relevant chain from chromatin remodelling to autophagy, oxidative‐stress control, epithelial homeostasis and colonic inflammation. SETD2 represents an intermediate stage in which intestinal inflammatory and autophagy–regulatory functions have been demonstrated separately but not yet mechanistically connected. DNA methylation, by comparison, is strongly associated with IBD at the epigenomic level, whereas its direct regulation of intestinal autophagy remains less well defined [37–39, 41, 44, 45, 49].

Taken together, these differences indicate that epigenetic regulation of autophagy in IBD should not be considered a single uniformly established mechanism. Instead, the field currently spans a continuum from disease‐supported pathways, such as BRG1‐dependent regulation of epithelial autophagy, to partially connected mechanisms such as SETD2, and to broader epigenetic alterations whose autophagic consequences remain largely unexplored. Defining where each pathway lies along this continuum may help prioritise future mechanistic studies and identify the epigenetic–autophagy interactions with the greatest potential for translational investigation.

### 4.4. Noncoding RNA Regulation of Autophagy in IBD

Noncoding RNAs constitute a multilayered post‐transcriptional regulatory network that can influence different stages of autophagy through target‐mRNA repression, modulation of transcript stability, interactions with RNA‐binding proteins and ceRNA networks. In IBD, these regulatory processes may affect epithelial stress adaptation, intracellular bacterial clearance, Paneth‐ and goblet‐cell function, inflammasome activity and mucosal barrier homeostasis [52, 53].

The mechanisms and levels of evidence differ substantially among noncoding RNA classes. miRNAs generally act through direct repression of specific autophagy‐related transcripts and currently have the most extensive support from human IBD tissues, experimental colitis and disease‐relevant cellular systems. Long noncoding RNAs (lncRNAs) and circular RNAs (circRNAs) more frequently function through ceRNA networks or interactions with RNA‐binding proteins. Several circRNA pathways have been functionally examined in intestinal epithelial cells and experimental colitis, whereas the causal relationship linking individual lncRNAs, autophagy and IBD phenotypes remains less completely established. The available evidence is therefore organised below according to RNA class, direction of autophagy regulation and degree of disease‐specific validation [52, 53].

#### 4.4.1. MicroRNAs

miRNAs are short noncoding RNAs that generally bind to complementary sequences within target mRNAs, resulting in translational repression or transcript degradation [52]. Because a single miRNA may regulate multiple targets and an individual autophagy gene may be controlled by several miRNAs, the biological effect of a miRNA cannot be inferred solely from its expression level. Its functional consequence depends on the autophagy component being targeted, the responding cell type, the microbial or inflammatory stimulus and whether the resulting autophagic response is adaptive or maladaptive in that setting.

##### 4.4.1.1. miRNAs That Suppress Autophagy and Antibacterial Defence

A well‐supported microbial–miRNA–autophagy pathway in CD involves CD‐associated AIEC. Nguyen et al. demonstrated that AIEC infection activates NF‐*κ*B signalling in intestinal epithelial cells and induces miR‐30c and miR‐130a. miR‐30c and miR‐130a repress ATG5 and ATG16L1, respectively, resulting in reduced autophagic activity, impaired intracellular bacterial clearance, increased AIEC persistence and enhanced inflammatory responses [25]. Consistent expression changes were observed in mouse enterocytes and ileal biopsy specimens from patients with CD, thereby connecting microbial exposure, miRNA induction, defective autophagy and disease‐relevant intestinal alterations.

This autophagy‐suppressive effect can extend beyond directly infected cells. AIEC‐infected intestinal epithelial cells release exosomes enriched in miR‐30c and miR‐130a. Transfer of these exosomes to recipient epithelial cells reduces ATG5 and ATG16L1 expression and impairs autophagy‐mediated AIEC clearance [26]. This mechanism indicates that local microbial infection may propagate an autophagy‐deficient state across the epithelial compartment through intercellular miRNA transfer.

miR‐106b and miR‐93 directly target ATG16L1 and suppress autophagy‐dependent bacterial clearance in epithelial cells. An inverse relationship between miR‐106b and ATG16L1 expression was also observed in intestinal tissues from patients with active CD [54]. Although this pathway produces a functional outcome similar to that of AIEC‐induced miR‐30c and miR‐130a, current evidence does not establish miR‐93 and miR‐106b as components of the same AIEC‐NF‐*κ*B response. These observations support miR‐106b and miR‐93 as post‐transcriptional regulators of ATG16L1, although the inverse association with ATG16L1 in human CD tissues has been demonstrated specifically for miR‐106b. An additional CD‐relevant mechanism involves miR‐196 and human IRGM. In inflamed intestinal epithelium from patients with CD, miR‐196 was reported to preferentially repress the protective human IRGM c.313C allele, whereas the disease‐associated c.313 T variant escaped this regulation [55]. This allele‐specific interaction impaired human IRGM‐dependent xenophagy and intracellular control of AIEC, providing a direct link between miRNA regulation, host genotype and antibacterial autophagy in CD.

miR‐130a‐3p further connects defective autophagy with inflammatory signalling. miR‐130a‐3p is increased in colonic tissues from patients with CD and promotes disease‐related inflammation by targeting ATG16L1 and modulating NF‐*κ*B signalling [56]. Genetic deletion of miR‐130a reduced the severity of trinitrobenzene sulfonic acid‐induced colitis, whereas pharmacological inhibition of miR‐130a‐3p induced autophagy, suppressed NLRP3 inflammasome activity in macrophages, and ameliorated experimental colitis [56]. The integration of patient tissue analysis, gain‐ and loss‐of‐function experiments and therapeutic intervention provides relatively strong preclinical evidence for miR‐130a‐3p as a regulator of the autophagy–inflammation interface in CD.

In UC, miR‐29a has been reported to suppress epithelial autophagy by directly targeting ATG9A, a membrane‐trafficking component of the autophagy machinery [57]. miR‐29a overexpression reduced LC3 puncta and the LC3‐II/LC3‐I ratio while increasing SQSTM1/p62 abundance, and restoration of ATG9A partially reversed these changes [57]. This finding suggests that IBD‐associated miRNAs may impair autophagy not only by targeting the ATG5–ATG12–ATG16L1 machinery but also by altering ATG9A‐dependent membrane delivery.

A subsequent large study of treatment‐naïve paediatric CD identified increased miR‐29 expression, particularly miR‐29b, as predictive of severe intestinal inflammation and stricturing within the study cohort and associated it with Paneth‐cell loss [58]. However, that study did not examine ATG9A or autophagic flux. It therefore extends the clinical and epithelial relevance of the miR‐29 family but does not independently validate the miR‐29a–ATG9A autophagy mechanism.

miR‐142‐3p can also directly repress ATG16L1. In cellular models, miR‐142‐3p mimics inhibited starvation‐ and muramyl‐dipeptide‐induced autophagy, whereas inhibition of miR‐142‐3p enhanced starvation‐induced autophagy [59]. Although these experiments establish a direct miR‐142‐3p–ATG16L1 relationship, the pathway has not been functionally linked to disease severity in a complete experimental colitis model or a sufficiently characterised human IBD cohort. It should therefore be regarded as an IBD‐relevant cellular mechanism rather than a fully established disease pathway.

Collectively, autophagy‐suppressing miRNAs in IBD converge on a limited number of critical nodes, particularly ATG5, ATG9A and ATG16L1 [25, 54, 57, 59]. Such convergence may amplify autophagy defects produced by genetic susceptibility, microbial exposure or inflammatory signalling. Nevertheless, several of these miRNAs also regulate NF‐*κ*B, NLRP3 or other inflammatory pathways, and their disease effects cannot always be attributed exclusively to altered autophagy [56, 60].

##### 4.4.1.2. miRNA‐Mediated Autophagy Activation and Context‐Dependent Effects

An increase in autophagy is not necessarily associated with mucosal protection. The functional outcome depends on whether the autophagic response restores cellular homeostasis or contributes to a maladaptive response to persistent microbial or inflammatory stress.

Extracellular vesicles derived from *Fusobacterium nucleatum* provide a disease‐relevant example. *F. nucleatum*‐derived extracellular vesicles reduced miR‐574‐5p expression in intestinal epithelial cells, thereby relieving miR‐574‐5p‐mediated repression of CARD3 and increasing autophagic flux [60]. This response was accompanied by proinflammatory cytokine production, loss of tight‐junction proteins, epithelial barrier dysfunction and aggravation of DSS‐induced colitis. The restoration of miR‐574‐5p, CARD3 depletion or chloroquine‐mediated inhibition of autophagy attenuated the inflammatory and barrier phenotypes [60]. Thus, in this setting, reduced miR‐574‐5p permits CARD3‐dependent autophagy activation that contributes to tissue injury rather than protection.

This pathway contrasts with the AIEC model, in which suppression of epithelial autophagy promotes intracellular bacterial persistence [25, 60]. Together, these findings demonstrate that the inflammatory significance of autophagy cannot be determined solely from whether LC3‐related activity increases or decreases. The microbial stimulus, molecular target, magnitude and duration of the autophagic response, and affected intestinal cell population must also be considered.

#### 4.4.2. Long Noncoding RNAs

lncRNAs regulate cellular processes through interactions with DNA, other RNAs, chromatin‐associated proteins, transcription factors and RNA‐binding proteins. Within the intestinal mucosa, lncRNAs can influence epithelial renewal, secretory‐cell function, junctional integrity and inflammatory signalling [53]. However, compared with miRNAs, relatively few studies have established a complete causal pathway linking a specific lncRNA, autophagy and an IBD phenotype.

H19 is the most directly studied intestinal autophagy‐related lncRNA. In mice exposed to septic stress, human intestinal tissues, primary intestinal organoids and cultured epithelial cells, increased H19 expression suppressed autophagy, impaired Paneth‐ and goblet‐cell function and disrupted intestinal barrier integrity [61]. H19 deletion enhanced epithelial autophagy and secretory‐cell function, whereas H19 overexpression inhibited rapamycin‐induced autophagy and diminished its barrier‐protective effect [61]. These findings establish H19 as a regulator of intestinal epithelial autophagy and barrier homeostasis, although the principal in vivo disease setting was septic intestinal injury rather than IBD.

A more recent study found increased H19 expression in patients with UC and in lipopolysaccharide‐stimulated HT‐29 cells [62]. H19 knockdown increased autophagy‐related activity, reduced apoptosis and improved cell survival under inflammatory stimulation [62]. This study provides a more direct association between H19, autophagy and UC‐related epithelial inflammation. Nevertheless, the functional autophagy experiments were performed predominantly in a cell‐based system, and an in vivo experiment directly testing autophagy dependence was not conducted.

The available evidence therefore supports H19 as a biologically plausible regulator connecting epithelial autophagy, secretory‐cell function and intestinal inflammation. However, the complete causal sequence from H19 dysregulation to autophagy impairment and IBD progression has not yet been established within a single in vivo disease model. Autophagy‐rescue or blockade experiments will be required to determine whether the inflammatory effects of H19 in UC are dependent on its regulation of autophagy [61, 62].

#### 4.4.3. Circular RNAs

circRNAs are covalently closed transcripts generated by back‐splicing. Their closed structure confers relative resistance to exonuclease degradation, and they can regulate gene expression by sequestering miRNAs, interacting with RNA‐binding proteins or participating in ceRNA networks. In IBD‐related studies, circRNAs have been linked to both suppression and activation of epithelial autophagy [53].

##### 4.4.3.1. Autophagy‐Suppressing and Potentially Pathogenic circRNAs

hsa_circRNA_103124 is increased in peripheral blood mononuclear cells from patients with CD and correlates with white blood cell counts and faecal calprotectin levels [63]. Mechanistically, hsa_circRNA_103124 acts as a sponge for miR‐650, increases AKT2 signalling and suppresses rapamycin‐induced autophagy in human intestinal epithelial cells and Caco‐2 cells. Knockdown and overexpression experiments, together with miR‐650 rescue, support a hsa_circRNA_103124–miR‐650–AKT2 regulatory pathway [63].

However, the autophagy component of this study was predominantly evaluated in cultured cells, while the human component established expression and clinical correlations rather than direct mucosal autophagy dysfunction. The pathway therefore provides disease‐associated mechanistic evidence but still requires validation in intestinal tissue and experimental colitis.

##### 4.4.3.2. Autophagy‐Promoting and Potentially Protective circRNAs

circGMCL1 represents a comparatively complete circRNA–autophagy mechanism in experimental chronic colitis. circGMCL1 acts as a ceRNA for miR‐124‐3p, thereby increasing ANXA7 expression and promoting epithelial autophagy [64]. Reduced circGMCL1 expression impaired autophagy, enhanced NLRP3 inflammasome‐mediated epithelial pyroptosis, increased inflammatory cytokine release and aggravated barrier injury. Conversely, delivery of circGMCL1 using poly (lactic‐co‐glycolic acid) (PLGA) microspheres restored autophagy, reduced pyroptosis and ameliorated colitis in IL‐10‐deficient mice [64]. The combination of human CD tissue analysis, epithelial‐cell experiments, autophagic‐flux assessment and in vivo intervention provides relatively strong preclinical support for this pathway.

circHECTD1 constitutes a parallel UC‐related mechanism. circHECTD1 and the RNA‐binding protein HuR (ELAVL1) were reduced in colonic mucosal tissues from patients with active UC, together with evidence of impaired autophagy [65]. Mechanistic experiments showed that circHECTD1 sequesters miR‐182‐5p, increases HuR expression, and promotes AMP‐activated protein kinase (AMPK)/mechanistic target of rapamycin (mTOR)‐dependent autophagy in lipopolysaccharide‐stimulated Caco‐2 cells. circHECTD1 overexpression also reduced inflammatory cytokine production and NLRP3 inflammasome activity and ameliorated colonic injury in DSS‐treated mice [65]. These findings link a human disease‐associated expression pattern to molecular interaction, cellular rescue and animal intervention, although the human tissue data remain associative.

A newer study identified reduced hsa_circ_0005255 expression in intestinal tissues from patients with CD and examined its function in epithelial cells and DSS‐induced colitis [66]. hsa_circ_0005255 acted through the miR‐23a‐3p–nuclear receptor coactivator 3 (NCOA3) axis to promote epithelial‐cell proliferation and autophagy, reduce apoptosis and inflammatory signalling and improve barrier‐related outcomes. Overexpression also attenuated DSS‐induced colonic injury [66]. This study extends the spectrum of potentially protective autophagy‐promoting circRNAs in CD‐associated research. Nevertheless, DSS‐induced colitis is not a CD‐specific model [67], and the patient sample size and cross‐model validation remain limited. The pathway should therefore be considered emerging rather than definitive disease‐specific evidence.

##### 4.4.3.3. Predicted circRNA–miRNA–autophagy networks

RNA sequencing of colon tissues from mice with DSS‐induced colitis identified differentially expressed circRNAs and generated a predicted chr10: 115386962–115390436+/mmu‐miR‐6914‐5p/Atg7 network [68]. The study performed preliminary expression validation of selected circRNAs but did not establish direct binding among the proposed RNAs, functional regulation of Atg7, altered autophagic flux or a causal effect on colitis.

This network therefore constitutes a hypothesis‐generating model rather than an established circRNA–autophagy pathway. Direct perturbation of the circRNA, confirmation of miRNA binding, target‐rescue experiments and functional assessment of autophagic flux and intestinal inflammation will be required to validate the proposed relationship.

#### 4.4.4. tRNA‐Derived Small RNAs

A recent study identified 5 ^′^‐tiRNA‐Lys as an intestinal epithelial tRNA‐derived small RNA that is increased in inflamed epithelial tissue from patients with IBD and can enhance epithelial autophagy in experimental systems [69]. Mechanistically, 5 ^′^‐tiRNA‐Lys bound EWSR1, disrupted the EWSR1‐Drosha‐DGCR8 microprocessor interaction, suppressed maturation of miR‐125a, increased the autophagy‐promoting target UVRAG and ameliorated DSS‐induced colitis in mice [69]. This pathway is directly relevant to the regulation–autophagy–IBD axis, but it should still be interpreted as an emerging mechanism requiring further validation across independent human cohorts and disease models.

#### 4.4.5. Comparative Interpretation and Remaining Questions

The evidence supporting noncoding RNA regulation of autophagy in IBD is not equally mature across RNA classes. miRNAs currently have the strongest target‐level evidence, with several studies identifying direct regulation of ATG5, ATG9A or ATG16L1 and demonstrating effects in patient tissues, intestinal epithelial cells, macrophages or experimental colitis [25, 54, 56–60]. circRNAs have yielded increasingly detailed epithelial mechanisms, including both autophagy‐suppressing pathways, such as hsa_circRNA_103124, and autophagy‐promoting pathways, such as circGMCL1, circHECTD1 and hsa_circ_0005255 [63–66]. However, most circRNA findings remain preclinical. For lncRNAs, H19 provides a compelling intestinal autophagy mechanism, but the autophagy‐dependent contribution of H19 to IBD progression has not yet been demonstrated conclusively in vivo.

A second distinction concerns the biological interpretation of autophagy direction. Suppression of ATG5‐, ATG9A‐ or ATG16L1‐dependent antibacterial autophagy generally compromises epithelial microbial clearance and defence [25, 54, 57, 59]. By contrast, autophagy activation may either preserve epithelial homeostasis or contribute to barrier injury, as illustrated by the opposing effects observed in the circGMCL1/circHECTD1 and *F. nucleatum*‐extracellular‐vesicle models [60, 64, 65]. Static changes in LC3, SQSTM1/p62 or an individual ATG protein are therefore insufficient to classify a noncoding RNA as protective or pathogenic [70].

Future studies should integrate direct noncoding RNA perturbation with dynamic autophagic‐flux measurements, lysosomal‐function assays, target‐rescue experiments and genetic or pharmacological tests of autophagy dependence [70]. Cell‐type‐specific analysis will also be essential because the consequences of altered autophagy may differ among enterocytes, Paneth cells, intestinal stem cells, macrophages and lymphocytes [4, 5]. Finally, validation in well‐characterised human CD and UC tissues, organoid systems, and multiple complementary disease models will be necessary to distinguish mechanisms that operate in human IBD from findings restricted to transformed cell lines or acute chemically induced colitis [67].

Representative epigenetic and post‐transcriptional regulators of autophagy discussed in this review, together with their disease context, experimental models, regulatory direction, IBD‐related consequences and evidence category, are summarised in Table 1.

**Table 1:** Representative classical epigenetic and post‐transcriptional regulators of autophagy discussed in this review and their relevance to inflammatory bowel disease.

Regulatory class	Regulator	Autophagy target/pathway	IBD context/model	Main effect on autophagy and IBD‐related phenotype	Evidence category	Evidence interpretation	Reference(s)
DNA methylation	IBD‐associated DNA methylation/DNMT‐dependent regulation	Autophagy‐related gene transcription (general; ATG5/LC3 and related machinery)	Human UC mucosa and circulating methylome; mechanistic autophagy evidence mainly from non‐IBD systems	IBD‐associated methylation changes are reproducible, but a direct methylation‐autophagy causal pathway in IBD remains unproven	Indirect non‐IBD mechanistic evidence	Human IBD association is strong, but the direct autophagy‐regulatory component remains indirect.	[37–42]
Histone modification	SETD2	H3K36me3; ATG12‐related autophagy	Experimental colitis; autophagy mechanism demonstrated in clear‐cell renal cell carcinoma	SETD2 modulates oxidative stress and intestinal inflammation; SETD2 deficiency impairs autophagy through altered ATG12 regulation in a non‐IBD model	Disease‐relevant experimental evidence	IBD inflammatory function and non‐IBD autophagy mechanism both exist, but their convergence in IBD remains unproven.	[43–45]
Histone modification	SIRT6	ULK1/PUMA‐associated autophagy regulation	Non‐IBD experimental systems	Promotes autophagy through interaction with ULK1 and competitive binding to PUMA	Indirect non‐IBD mechanistic evidence	Mechanistic autophagy evidence without IBD validation.	[46]
Chromatin remodelling	BRG1/SMARCA4	ATG16L1, AMBRA1, ATG7, WIPI2	Intestinal epithelial cells and experimental colonic inflammation	Promotes transcription of multiple autophagy genes, limits ROS accumulation, preserves epithelial homeostasis, and attenuates colonic inflammation	Disease‐relevant experimental evidence	Disease‐relevant mechanistic evidence in intestinal epithelial and experimental colitis models.	[49]
Chromatin remodelling	CHD6	Autophagy and DNA‐damage‐response programmes	Non‐IBD cellular systems	Coordinates autophagy with genome‐stress responses	Indirect non‐IBD mechanistic evidence	Candidate extension without IBD‐specific validation.	[50]
miRNA	miR‐30c/miR‐130a	ATG5/ATG16L1	AIEC‐infected intestinal epithelial cells; mouse enterocytes; ileal biopsies from CD; exosomal transfer	Suppress antibacterial autophagy, increase intracellular AIEC persistence and inflammation; exosomes propagate the autophagy‐deficient state	Direct IBD evidence	Human CD tissue association plus disease‐relevant cellular and microbial mechanism.	[25, 26]
miRNA	miR‐106b/miR‐93	ATG16L1	Intestinal epithelial cells; active CD tissue association demonstrated for miR‐106b	Suppress ATG16L1‐dependent autophagy and bacterial clearance	Direct IBD evidence	Direct target‐level cellular evidence with partial human CD validation.	[54]
miRNA	miR‐196	Human IRGM (allele‐specific regulation)	Inflamed intestinal epithelium from patients with CD	Preferential repression of the protective human IRGM c.313C allele impairs human IRGM‐dependent xenophagy and intracellular AIEC control	Direct IBD evidence	Direct human CD genotype‐miRNA‐xenophagy mechanism.	[55]
miRNA	miR‐130a‐3p	ATG16L1; NF‐*κ*B/NLRP3‐related signalling	CD tissues; macrophages; TNBS‐induced colitis	Suppresses autophagy and promotes inflammatory signalling; inhibition restores autophagy, suppresses NLRP3 activity and ameliorates colitis	Direct IBD evidence	Human association plus in vivo functional intervention.	[56]
miRNA	miR‐29a/miR‐29 family	ATG9A (miR‐29a); clinical phenotype association for miR‐29 family	UC epithelial models; treatment‐naive paediatric CD cohort	MiR‐29a suppresses epithelial autophagy via ATG9A; increased miR‐29 is associated with severe paediatric CD and Paneth‐cell loss, but the CD cohort did not validate the ATG9A mechanism	Disease‐relevant experimental evidence	Direct UC cellular mechanism plus independent human CD association; complete cross‐context causal chain remains incomplete.	[57, 58]
miRNA	miR‐142‐3p	ATG16L1	Cellular autophagy models	Directly represses ATG16L1 and inhibits starvation‐ and MDP‐induced autophagy	Indirect non‐IBD mechanistic evidence	Direct target‐level cellular evidence, but no complete colitis model or sufficiently characterised human IBD cohort.	[59]
miRNA	miR‐574‐5p	CARD3	*F. nucleatum* extracellular vesicles; intestinal epithelial cells; DSS colitis	Loss of miR‐574‐5p releases CARD3, increases autophagic flux and, in this context, aggravates inflammatory and barrier injury	Disease‐relevant experimental evidence	Disease‐relevant mechanistic and in vivo evidence; autophagy is contextually pathogenic.	[60]
lncRNA	H19	Epithelial autophagy; Paneth/goblet‐cell function	Septic‐stress mice, human intestinal tissues, organoids and epithelial cells; UC tissues and LPS‐stimulated HT‐29 cells	Increased H19 suppresses epithelial autophagy and impairs secretory‐cell/barrier function; H19 knockdown enhances autophagy in inflammatory epithelial models	Disease‐relevant experimental evidence	Strong intestinal mechanism; complete autophagy‐dependent IBD causal chain not yet established.	[61, 62]
circRNA	hsa_circRNA_103124	miR‐650/AKT2	CD PBMCs; intestinal epithelial cells and Caco‐2 cells	Increased circRNA_103124 suppresses rapamycin‐induced autophagy through the miR‐650/AKT2 axis	Direct IBD evidence	Human disease association plus cellular mechanism; mucosal autophagy validation remains incomplete.	[63]
circRNA	circGMCL1	miR‐124‐3p/ANXA7	Human CD tissue; epithelial cells; IL‐10‐deficient chronic colitis; PLGA‐mediated delivery	Promotes epithelial autophagy, suppresses NLRP3‐associated pyroptosis and improves barrier injury and colitis	Direct IBD evidence	Human tissue association with epithelial‐cell, autophagic‐flux, and in vivo intervention evidence.	[64]
circRNA	circHECTD1	miR‐182‐5p/HuR; AMPK/mTOR	Active UC mucosa; LPS‐stimulated Caco‐2 cells; DSS colitis	Promotes enterocyte autophagy, reduces inflammatory cytokines and NLRP3 activity, and ameliorates colonic injury	Direct IBD evidence	Human association plus cellular/in vivo functional evidence; human tissue data remain associative.	[65]
circRNA	hsa_circ_0005255	miR‐23a‐3p/NCOA3	CD intestinal tissue; epithelial cells; DSS colitis	Promotes epithelial‐cell proliferation and autophagy, reduces apoptosis/inflammatory signalling, and attenuates colonic injury	Disease‐relevant experimental evidence	Emerging disease‐associated mechanism; DSS is not CD‐specific.	[66, 67]
circRNA	Predicted circRNA‐miR‐6914‐5p‐Atg7 network	Atg7 (predicted)	Colon RNA‐seq from DSS‐induced colitis	Bioinformatic network predicts circRNA‐miRNA‐Atg7 regulation, but direct binding, autophagic‐flux effects and causal disease relevance were not established	Hypothesis‐generating evidence	Computational or predicted network retained only as a hypothesis‐generating model.	[68]
tRNA‐derived small RNA	5 ^′^‐tiRNA‐Lys	EWSR1‐miR‐125a‐UVRAG axis	Inflamed intestinal epithelium from patients with IBD; intestinal epithelial cells; DSS colitis	Binds EWSR1, suppresses miR‐125a maturation, increases UVRAG expression, enhances epithelial autophagy, and ameliorates DSS colitis	Direct IBD evidence	Recent human‐expression, target‐level, cellular, and in vivo evidence; independent validation is still needed.	[69]

*Note:* Evidence categories: Direct IBD evidence, evidence from human IBD samples or patient‐derived data linked to a defined regulator‐autophagy relationship, with target‐level or functional autophagy support in human or disease‐relevant experimental systems; this category indicates direct human‐disease relevance and does not necessarily imply that the complete causal pathway has been functionally demonstrated in primary human tissue. Disease‐relevant experimental evidence, evidence from experimental colitis or IBD‐relevant epithelial/immune‐cell systems without complete human mechanistic validation; Indirect non‐IBD mechanistic evidence, regulator‐autophagy evidence derived mainly from non‐IBD systems or general cellular models; Hypothesis‐generating evidence, computational or predicted networks without sufficient experimental validation. These categories describe evidentiary context rather than formal quality grades.

Abbreviations: AIEC, adherent‐invasive Escherichia coli; AKT2, AKT serine/threonine kinase 2; AMBRA1, autophagy and beclin 1 regulator 1; AMPK, AMP‐activated protein kinase; ANXA7, annexin A7; ATG, autophagy‐related; BRG1, Brahma‐related gene 1; Caco‐2, human colorectal adenocarcinoma cell line; CARD3, caspase recruitment domain family member 3; CD, Crohn’s disease; CHD6, chromodomain helicase DNA‐binding protein 6; circRNA, circular RNA; DGCR8, DiGeorge syndrome critical region 8; DNMT, DNA methyltransferase; DSS, dextran sulphate sodium; EWSR1, EWS RNA binding protein 1; H3K36me3, trimethylation of histone H3 lysine 36; HT‐29, human colorectal adenocarcinoma epithelial cell line; HuR, human antigen R/ELAVL1; IBD, inflammatory bowel disease; IEC, intestinal epithelial cell; IL‐10, interleukin‐10; IRGM, immunity‐related GTPase M; LC3, microtubule‐associated protein 1 light chain 3; lncRNA, long noncoding RNA; LPS, lipopolysaccharide; MDP, muramyl dipeptide; miRNA, microRNA; mTOR, mechanistic target of rapamycin; NCOA3, nuclear receptor coactivator 3; NF‐*κ*B, nuclear factor‐*κ*B; NLRP3, NLR family pyrin domain containing 3; PBMC, peripheral blood mononuclear cell; PLGA, poly (lactic‐co‐glycolic acid); PUMA, p53 upregulated modulator of apoptosis; RNA‐seq, RNA sequencing; ROS, reactive oxygen species; SETD2, SET domain containing 2 histone lysine methyltransferase; SIRT6, sirtuin 6; SMARCA4, SWI/SNF‐related matrix‐associated actin‐dependent regulator of chromatin subfamily A member 4; SQSTM1, sequestosome 1; tiRNA, tRNA‐derived stress‐induced RNA; TNBS, trinitrobenzene sulfonic acid; UC, ulcerative colitis; ULK1, Unc‐51‐like autophagy activating kinase 1; UVRAG, UV radiation resistance‐associated gene; WIPI2, WD repeat domain phosphoinositide‐interacting protein 2.

## 5. Translational Implications of Epigenetic and Post‐Transcriptional Regulation of Autophagy in IBD

The reversible and therapeutically modifiable nature of both classical epigenetic and post‐transcriptional regulation suggests that selected regulator‐autophagy pathways may provide therapeutic targets, biomarkers and tools for patient stratification in IBD. In principle, pathogenic noncoding RNAs could be inhibited, protective RNAs could be replaced or enhanced, abnormal DNA methylation or histone acetylation could be modified, and related molecular signatures could be used to identify patients with distinct biological abnormalities [9, 10, 71].

However, epigenetic and post‐transcriptional regulators, as well as autophagy‐related proteins, are widely expressed and frequently exert pleiotropic effects. The same molecular intervention may therefore produce different or even opposing effects in intestinal epithelial, immune and stromal cells. The translational objective should not be the non‐selective activation or inhibition of autophagy, but rather the identification and correction of disease‐, tissue‐ and cell‐specific regulatory abnormalities whose pathological effects are demonstrably dependent on altered autophagy [4, 5, 71].

### 5.1. RNA‐Based Therapeutic Strategies

miRNAs and other noncoding RNAs may be therapeutically manipulated using chemically modified antisense oligonucleotides, antagomirs, miRNA mimics, small‐molecule modulators or nucleic acid delivery systems. The direction of intervention should be determined by the underlying molecular mechanism. Pathogenic miRNAs that suppress protective antibacterial autophagy may require inhibition, whereas miRNAs or circRNAs that maintain epithelial autophagy and barrier homeostasis may require replacement or enhanced expression [71].

Preclinical studies provide direct mechanistic support for this strategy. Inhibition of miR‐130a‐3p enhanced autophagy, reduced NLRP3 inflammasome activity in macrophages and ameliorated experimental colitis [56]. Similarly, delivery of circGMCL1 using PLGA microspheres enhanced intestinal epithelial autophagy, suppressed NLRP3‐associated pyroptosis and improved colitis and barrier injury in IL‐10‐deficient mice [64]. These studies connect noncoding RNA intervention, restoration of autophagy and improvement of disease phenotypes, although their therapeutic efficacy has not yet been established in patients with IBD.

Obefazimod provides a clinically more advanced example of pharmacological miRNA modulation. This orally administered small molecule increases the expression of the anti‐inflammatory miR‐124 and has been evaluated in a phase 2b randomised induction trial and long‐term open‐label maintenance studies in patients with moderately to severely active UC [72, 73]. These studies demonstrate that pharmacological modulation of a defined miRNA can progress into clinical development.

Nevertheless, miR‐124 regulates multiple immune and inflammatory pathways [71–73], and the clinical activity of obefazimod has not been shown to depend on autophagy. Obefazimod should therefore be regarded as evidence for the clinical feasibility of miRNA‐directed pharmacology rather than as a validated autophagy‐dependent therapy.

Further development of RNA‐based treatments will depend on effective delivery. PLGA microspheres, lipid or polymeric nanoparticles, exosome‐like carriers and inflammation‐responsive materials may improve RNA stability, local retention and tissue‐specific release. However, carrier immunogenicity, batch consistency, endosomal escape, biodistribution, off‐target accumulation and long‐term toxicity require systematic evaluation. Future strategies will need to progress beyond general intestinal localisation towards selective delivery to defined epithelial or immune‐cell populations [71].

### 5.2. DNMT‐ and HDAC‐Related Therapeutic Strategies

DNMT inhibitors can reverse methylation‐associated transcriptional repression. However, currently available agents generally induce broad DNA hypomethylation and may affect genomic stability, cellular proliferation and immune‐cell differentiation. These non‐selective effects limit the rationale for systemic DNMT inhibition in a chronic non‐malignant disorder such as IBD [36, 40].

Some IBD‐related studies have instead used transient ex vivo epigenetic conditioning. Pretreatment of human mesenchymal stromal cells with 5‐azacytidine enhanced their migratory and immunomodulatory properties and improved their therapeutic activity in experimental colitis [74]. However, this approach represents the ex vivo modification of a cellular product rather than systemic DNMT inhibition in the patient. The study also did not establish that altered autophagy mediated the improved therapeutic effect.

These findings therefore support the feasibility of DNMT modulation in cell engineering but do not provide direct evidence for DNMT‐targeted correction of autophagy in IBD. Compared with non‐selective systemic demethylation, transient ex vivo conditioning or locus‐specific epigenome editing may offer a more plausible future direction.

Histone deacetylase (HDAC) inhibitors have generated more extensive pharmacological evidence in experimental intestinal inflammation. The selective HDAC6 inhibitor CKD‐506 suppressed NF‐*κ*B signalling and proinflammatory cytokine production in intestinal epithelial cells and macrophages and ameliorated acute DSS colitis, chronic colitis in IL‐10‐deficient mice and adoptive T‐cell transfer colitis [75]. In addition, reactive oxygen species‐responsive thioketal nanoparticles have been developed to release the HDAC6 inhibitor tubastatin A preferentially within inflamed colonic tissue, thereby enhancing local anti‐inflammatory activity while reducing exposure of non‐target tissues [76].

These studies support the preclinical feasibility of selective HDAC inhibition and colon‐targeted delivery. Nevertheless, their anti‐inflammatory effects cannot automatically be attributed to correction of an autophagy defect. HDAC isoforms regulate not only histone acetylation but also inflammatory transcription, cytoskeletal remodelling, protein homeostasis and autophagosome–lysosome function.

The establishment of an HDAC–autophagy–IBD pathway will therefore require dynamic autophagic‐flux measurements, direct target‐rescue experiments and genetic or pharmacological tests of autophagy dependence [70, 77]. Because individual HDAC isoforms may exert different effects in epithelial, myeloid and lymphoid cells, isoform selectivity and local delivery will be important prerequisites for further translation.

### 5.3. Epigenetic and Post‐Transcriptional Biomarkers and Patient Stratification

Epigenetic and post‐transcriptional signatures may have applications in diagnosis, prognosis, prediction of treatment response and pharmacodynamic monitoring. Candidate biomarkers include blood‐, saliva‐ or mucosa‐derived miRNAs and circRNAs, DNA methylation signatures, histone‐modification patterns and the expression of regulated ATGs [9].

DNA methylation has provided relatively direct human evidence for treatment‐response prediction. The EPIC‐CD study used peripheral‐blood samples obtained before biologic therapy in adults with CD to develop and externally evaluate DNA methylation signatures associated with treatment response [78]. Models developed for vedolizumab and ustekinumab showed external predictive performance, whereas the adalimumab model was not successfully validated in the external cohort. These findings suggest that epigenetic biomarkers may be treatment‐specific rather than universal predictors of biological response.

Importantly, the methylation signatures identified in EPIC‐CD were not shown to be directly related to autophagy. This study therefore demonstrates the clinical potential of epigenetic biomarkers for patient stratification rather than establishing a validated autophagy‐related biomarker.

miRNAs and circRNAs may similarly reflect disease activity, disease behaviour or epithelial dysfunction. For example, miR‐29, circGMCL1 and circHECTD1 have been associated with specific IBD phenotypes or tissue alterations [58, 64, 65]. A recent salivary exosome‐derived miRNA study reported an eight‐miRNA signature that distinguished patients with IBD from healthy controls, with five miRNAs showing strong correlations with disease activity [79]. However, that salivary signature was not shown to be an autophagy biomarker and should be interpreted as general RNA‐biomarker evidence rather than validation of an autophagy‐related biomarker. Expression associations may also be influenced by inflammatory activity, medication exposure, age, smoking, nutritional status and the cellular composition of the analysed sample [38, 78, 79].

Candidate biomarkers should therefore be evaluated in independent cohorts and compared with established measures, including C‐reactive protein, faecal calprotectin and endoscopic indices [78], to demonstrate incremental clinical value. For the mechanisms discussed in this review, composite biomarkers integrating an epigenetic or post‐transcriptional regulator, its validated autophagy target, and a clinically relevant phenotype may be more informative than measurement of a single molecule. For example, the relative expression of a miRNA and ATG16L1, or a molecular signature reflecting defective antibacterial autophagy, may theoretically provide greater mechanistic specificity than an isolated miRNA measurement. However, no composite regulator‐autophagy biomarker has yet been validated for routine clinical use in IBD.

### 5.4. Delivery, Tissue and Cell‐Type Specificity, and Safety

The principal technical obstacles to RNA‐based and epigenetic therapies include nucleic acid degradation, insufficient tissue uptake, inefficient endosomal escape, rapid systemic clearance, unintended biodistribution and unintended immune activation. Viral vectors or persistent epigenetic interventions may additionally produce prolonged and difficult‐to‐reverse effects. Delivery systems must therefore control not only the intestinal location of the intervention but also its duration, dose and cellular destination [71].

Tissue targeting and cell‐type targeting represent distinct translational requirements. Oral, rectal, pH‐responsive, reactive oxygen species‐responsive and microbiota‐responsive systems may increase drug accumulation within a specific intestinal segment or inflamed region, but they do not necessarily ensure uptake by the intended cell population [76, 80].

The same autophagy‐directed intervention may have different consequences in enterocytes, Paneth cells, macrophages, T cells or fibroblasts. Restoration of antibacterial autophagy in epithelial cells may promote intracellular microbial clearance, whereas increasing autophagy in activated immune cells or fibrosis‐associated stromal cells may not produce the same therapeutic outcome [4, 5, 26, 27]. Future delivery systems should therefore combine intestinal localisation with cell‐selective ligands, disease‐microenvironment‐responsive materials or local administration to achieve both tissue and cellular specificity.

Safety assessment must also account for the bidirectional functions of autophagy. Excessive inhibition may impair microbial clearance, antigen processing and epithelial repair, whereas persistent or excessive activation may support abnormal cell survival or maladaptive stress responses. The therapeutic objective should be restoration of an appropriate level of autophagy in a defined disease stage and cellular context rather than global activation or suppression of the autophagy machinery [4, 5].

### 5.5. Disease‐Specific Considerations in CD and UC and Requirements for Future Translation

CD and UC differ substantially in anatomical distribution, depth of inflammation, and complications and should therefore not be approached using identical epigenetic‐ or RNA‐mediated autophagy strategies.

UC is primarily confined to the colon and is characterised by continuous mucosal inflammation. These features make rectal administration, colon‐targeted oral delivery and repeated mucosal sampling comparatively feasible. Strategies aimed at restoring epithelial barrier function, regulating inflammasome activity or delivering locally acting RNA‐ or HDAC‐directed treatments may therefore be particularly suitable for tissue targeting and pharmacodynamic evaluation in UC.

CD can affect different regions of the gastrointestinal tract, frequently involves the terminal ileum and may present with discontinuous and transmural inflammation, fibrosis, strictures and fistulas. Ileal Paneth‐cell dysfunction, NOD2–ATG16L1‐associated abnormalities in microbial handling, and persistent AIEC colonisation may further create distinct therapeutic requirements [13, 25–27, 29].

A formulation released only within the colonic lumen may be insufficient for ileal, transmural or fibrotic disease. Some patients with CD may require segment‐specific release, deeper tissue penetration or systemic delivery directed towards selected epithelial, myeloid or stromal populations.

Disease‐specific interpretation should also guide experimental‐model selection. DSS‐induced colitis is useful for investigating acute epithelial injury and colonic mucosal inflammation but does not adequately reproduce the ileal involvement, transmural inflammation, fibrosis or penetrating complications of CD. Therapeutic effects demonstrated only in DSS‐treated mice should therefore not be described as CD‐specific evidence without validation in complementary genetic, immune‐mediated, microbial or chronic fibrosis models [67].

Future translation should follow a staged evidence framework. Direct molecular interactions and target‐rescue effects should first be demonstrated. Altered autophagy should then be confirmed using dynamic flux measurements rather than static LC3 or SQSTM1/p62 abundance alone [70]. The inflammatory or barrier phenotype should also be shown to depend on the proposed autophagy pathway through genetic or pharmacological interruption.

The mechanism should subsequently be localised to a defined cell population and validated in human CD or UC tissues, primary cells or patient‐derived organoids. Therapeutic development will further require disease‐appropriate animal models, pharmacokinetic and biodistribution studies, repeated‐dose toxicity assessment and evaluation alongside current standards of care. Early clinical trials should include paired mucosal and peripheral‐blood sampling to determine whether the intervention produces the intended epigenetic or post‐transcriptional target engagement together with the expected autophagic effect within the target intestinal compartment.

Overall, epigenetic and post‐transcriptional regulation of autophagy provides a biologically plausible framework for precision treatment and patient stratification in IBD. Nevertheless, the strongest current evidence supports the preclinical feasibility of manipulating selected noncoding RNA‐autophagy pathways and the broader clinical feasibility of epigenetic or miRNA modulation. No specific epigenetic‐ or RNA‐mediated autophagy therapy or composite biomarker has yet been validated for routine IBD care. Successful translation will require disease‐specific, tissue‐ and cell‐type‐resolved strategies that adequately address delivery, safety, target engagement and autophagy dependence.

## 6. Conclusion and Future Perspectives

Autophagy is an important mechanism linking intestinal epithelial homeostasis, antimicrobial defence, immune regulation, microbial interactions and cellular stress responses in IBD. Accumulating evidence indicates that classical epigenetic mechanisms, including DNA methylation, histone modifications and chromatin remodelling, and post‐transcriptional mechanisms mediated by noncoding RNAs can influence the expression or activity of ATGs and thereby contribute to intestinal inflammation. Collectively, these studies support epigenetic and post‐transcriptional regulation of autophagy as biologically relevant components of IBD pathogenesis. However, the maturity of the evidence differs substantially among regulatory mechanisms. Several miRNA‐, circRNA‐ and tiRNA‐related pathways are supported by direct target validation and functional studies in experimental colitis, whereas many proposed mechanisms involving DNA methylation, histone modifications, chromatin‐remodelling factors and lncRNAs remain based on associative or indirect evidence, including observations from non‐IBD models.

An important limitation is that mechanistic evidence from human IBD remains relatively limited. Much of the current understanding has been derived from cell lines and experimental models, whereas studies in patients are frequently based on cross‐sectional measurements of peripheral blood or intestinal biopsies. Such approaches may not fully capture the heterogeneity of CD and UC with respect to disease location, inflammatory activity, disease behaviour and treatment exposure. Moreover, bulk tissue analyses do not distinguish whether an observed regulatory alteration arises from molecular reprogramming within a specific cell population or simply reflects changes in cellular composition. This distinction is particularly important because the same epigenetic or post‐transcriptional regulator, or the same autophagy pathway, may exert different functions in epithelial, immune and stromal compartments. Greater use of well‐characterised human cohorts together with single‐cell and spatially resolved analyses should help define where disease‐associated regulator‐autophagy alterations occur and whether they are reproducible across patient populations.

A further challenge is the distinction between association and causality. The coexistence of a regulatory alteration, altered expression of an ATG, and intestinal inflammation does not by itself establish a causal regulator‐autophagy‐IBD pathway. Similarly, static measurements of LC3, SQSTM1/p62, ATG expression or autophagosome abundance cannot fully define autophagic activity [70]. Future mechanistic studies should therefore move beyond descriptive associations and combine dynamic assessment of autophagy with functional perturbation and rescue approaches. Demonstrating that disruption or restoration of the proposed autophagy pathway correspondingly modifies the inflammatory or epithelial phenotype will be particularly important for identifying mechanisms with genuine disease relevance.

The molecular heterogeneity of IBD is also unlikely to be explained by a single epigenetic or post‐transcriptional alteration, or by a single ATG. Integrated multi‐omics approaches provide an opportunity to place individual observations within broader regulatory networks. Combining epigenomic, transcriptomic, chromatin‐accessibility, proteomic, metabolomic, microbiome, genetic and clinical data may help distinguish primary regulatory mechanisms from secondary consequences of inflammation. Single‐cell and spatially resolved approaches will be especially valuable when integrated with these datasets because they can assign molecular changes to defined cell populations and anatomical niches. Longitudinal studies will further be required to clarify the temporal relationships among regulatory alterations, autophagy dysfunction, disease activity and therapeutic response.

Overall, epigenetic and post‐transcriptional regulation of autophagy provides a coherent framework for understanding an important component of IBD biology and has emerging implications for mechanism‐based therapies and patient stratification. At the same time, only a subset of proposed pathways has been validated across human samples, defined cell populations and functional autophagy assays. Progress in this field will therefore depend on integrating human validation, cell‐type‐resolved analysis, functional evidence of causality and multi‐omics approaches. Together, these approaches may enable the field to move from descriptive molecular associations towards reproducible disease mechanisms and, ultimately, clinically relevant biomarkers and therapeutic targets.

## Author Contributions

Yujun Liu and Wenliang Jiang contributed equally to this work.

## Funding

This study was supported by General Program of the Jiangsu Commission of Health, Grant M2025006.

## Conflicts of Interest

The authors declare no conflicts of interest.

## Data Availability

Data sharing not applicable to this article as no datasets were generated or analysed during the current study.
